# Manipulating Individual State During Migration: Carry‐Over Effects of Cumulative Stress on Survival

**DOI:** 10.1002/ece3.71812

**Published:** 2025-07-27

**Authors:** Ilona P. Grentzmann, Gilles Gauthier, Frédéric Angelier, Joël Bêty, Frédéric LeTourneux, Pierre Legagneux

**Affiliations:** ^1^ Département de Biologie and Centre d'études nordiques, Pavillon Vachon Université Laval Québec Québec Québec Canada; ^2^ Centre d'Études Biologiques de Chizé Centre National de Recherche scientifique Chizé France; ^3^ Département de Biologie and Centre d'études nordiques Université du Québec à Rimouski Québec Québec Canada; ^4^ Department of Natural Resource Sciences McGill University Sainte‐Anne‐de‐Bellevue Québec Canada

**Keywords:** body condition, capture–mark–recapture analyses, corticosterone, experimental design, snow geese

## Abstract

The stress response is a mechanism to cope with unpredictable events and minimize immediate threats to survival. However, cumulated stress due to multiple stressors can have long‐term deleterious effects on fitness by impairing reproduction and survival. This aspect of stress physiology and its consequences on demographic traits have received little attention in wild populations, and such studies are mostly observational. Here, we investigate the demographic consequences of multiple stressors (fasting and prolonged captivity) experimentally imposed during spring migration on greater snow geese (*Anser caerulescens atlantica*). In 2009, female snow geese were captured at a spring staging site and kept in captivity for up to 4 days with or without access to food. Blood samples were taken at capture, banding, and release to measure corticosterone (CORT) levels, a stress‐response hormone, during the experiment. CORT response peaked within the first hours after capture and decreased during the following days in captivity. We observed that stress‐induced CORT levels of captive individuals at release depended on their pre‐experiment body condition, but not the stress‐induced peak CORT response. We showed no link with subsequent reproductive success, but we detected a negative carry‐over effects of food deprivation on survival in the following year. Pre‐treatment spring body condition and stress‐induced CORT levels had marginal effects on survival. We showed that cumulated stressors could have carry‐over effects on survival and that the intensity of the hormonal response can ultimately affect survival.

## Graphical abstract

Our field experiment examines the physiological consequences (stress hormone and body condition) of a prolonged stress on survival. A single prolonged stress event that occurs during a pivotal period (pre‐migration fattening period) can have lasting effects over several months impacting adult survival, a key demographic trait.

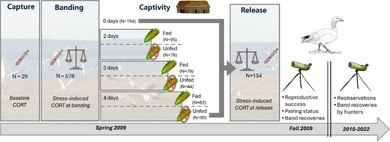

## 1 Introduction

In preparation for energy‐demanding periods of the life cycle, like winter, migration or reproduction, individual behaviour and physiology are adjusted accordingly (Jacobs and Wingfield 2000; Wingfield 2008). Individuals capable of optimally managing energy allocation prior to reproduction are predicted to maximise their fitness (Hennin et al. 2016; Bêty et al. 2003; Rowe et al. 1994). Effective resource allocation involves a dynamic balance between factors like initial body condition or individual quality (Galgani and Ravussin 2008), resource availability and acquisition, fattening rate, and costs such as competition for resources and predation risk (Wingfield and Kitaysky 2002).

The total energy requirements during predictable daily or seasonal activities is called the allostatic load (Romero et al. 2009; McEwen and Wingfield 2003). The normal variations within the allostatic load can encompass temporary increases in energy demand in response to an unpredictable event that quickly return to homeostasis (Romero et al. 2009). However, the organism can experience an allostatic overload when the demand in energy exceeds the available resources or when the increased demand is prolonged in time (McEwen and Wingfield 2003). For example, an accumulation of stressors can considerably increase the overall energy demand and extend the effects of stress over time, pushing individuals into a state of allostatic overload (Landys et al. 2006; McEwen and Wingfield 2003). In long‐lived species, a typical mechanism to cope with allostatic overload during the reproductive season is to reduce reproductive effort or even skip or abandon reproduction to save energy and allocate it to self‐maintenance and subsequent reproductive events (‘the prudent parent hypothesis’, Stearns 1992). Variation in breeding effort or breeding propensity is most pronounced when environmental conditions are unfavourable and limit resources required for breeding (Grandmont et al. 2023; Warren et al. 2014; Legagneux et al. 2012). However, a reduced breeding effort may not be sufficient to maintain survival when stressors are too intense or numerous (Romero and Wikelski 2001). Under such environmental stress, individuals may remain under a state of allostatic overload, which is associated with detrimental changes in behaviour, body condition or immunity, and consequently, with an increased vulnerability to predation or disease (O'Connor et al. 2014; Harrison et al. 2011).

Among the physiological drivers of behavioural and physiological adjustments to the environment, variations in glucocorticoids (GCs) are key for the maintenance of metabolism and energy storage (Romero et al. 2009). GCs are highly conserved hormones across vertebrates, secreted by the adrenal glands through the activation of the hypothalamic–pituitary–adrenal axis (HPA axis). Their role depends on the amount secreted (Sopinka et al. 2015; Romero 2004). The baseline level of GCs mediates daily energetic balance, foraging behaviour, body mass and metabolism (Landys et al. 2006). However, when an unpredictable acute stress occurs, the organism enters an emergency state (Angelier and Wingfield 2013; Wingfield et al. 1998) and secretes high amounts of GCs (Romero 2004). This initiates strong behavioural and physiological changes (Romero 2004; Wingfield and Kitaysky 2002) that go beyond the predictable variations based on life history strategy and available energy (Schoenle et al. 2018). Eventually, the secretion of GCs is downregulated by negative feedback, which keeps GC levels from becoming toxic (Romero 2004).

The role of GCs as a link between the state of the organism and its environment has been increasingly investigated in recent years, with several studies looking at the association between GC response to various stressors and fitness traits (Schoenle et al. 2018, 2021; Breuner et al. 2008). In addition, the role of GCs in mediating transitions between life‐history stages has also received a growing interest (Wingfield 2008). Recent meta‐analyses have linked both baseline and stress‐induced GC levels to variations in survival and reproductive success, but these relationships depend on the physiological and environmental context, the trait studied and the life history of the species (Schoenle et al. 2018, 2021; Sorenson et al. 2017; Hau et al. 2010; Bókony et al. 2009). Furthermore, sustained elevated GC concentrations can result in cumulative damage over time (McEwen and Seeman 1999), causing long‐lived animals to suffer greater costs of high GCs than short‐lived ones (Schoenle et al. 2018). However, the effect of increased GCs in response to multiple stressors at the intra‐specific level is less clear and varies with the energy‐demanding period considered (Cornelius et al. 2011). In birds, the main GC is corticosterone (CORT), and variations in its baseline level have been shown to prepare individuals for demanding periods in their life cycles (Romero 2002, 2004). For example, in migratory passerines, the increased energy period before migration (*Zugunruhe*) is linked with increased levels of CORT (Cornelius et al. 2013). Body condition is also a flexible component of the phenotype that can act as a buffer for energetically demanding life history stages and can be modified through CORT‐induced modifications in behaviour and diet (Hoarau et al. 2022; Angelier et al. 2007).

Allostatic overload caused by cumulative or prolonged stressors can have multiple effects, including an extended disruption of the GC response and feedback mechanisms (Romero 2004; Romero and Wikelski 2001) that can affect survival or reproduction at subsequent stages of the annual cycle (Harrison et al. 2011). Such carry‐over effects have been neglected in the literature but are increasingly studied (Harrison et al. 2011; Grandmont et al. 2023). Energy has been hypothesised to be the major link between life‐history stages affected by carry‐over effects (Legagneux et al. 2012; Harrison et al. 2011). Because of the functional link between energy regulation and GCs, GCs also have the potential to be key mediators of carry‐over effects (Legagneux et al. 2012; Schultner et al. 2014). Although carry‐over effects have been reported in many taxa (O'Connor et al. 2014), migrating birds are highly relevant models to study them because migration acts as a clear separation between life cycle stages (Harrison et al. 2011). Like classic stress responses, stress‐induced carry‐over effects are life‐history dependent and their links to fitness traits are increasingly studied (Schoenle et al. 2018, 2021). For example, several studies have reported a reduction of reproductive output several months after exposure to stressors in wild vertebrates (Grandmont et al. 2023; Harrison et al. 2011). However, the consequences of prolonged or accumulated stressors on survival remain poorly known and difficult to study, especially in long‐lived birds, because it requires following individuals over multiple years.

Field experiments are especially suitable to understand carry‐over effects resulting from cumulative stressors and the associated physiological mechanisms (Harrison et al. 2011). We carried out such an experiment on the greater snow goose (*Anser caerulescens atlantica*), a long‐lived migratory bird for which we have extensive long‐term monitoring data. We applied multiple stressors during spring staging by maintaining birds in captivity with or without access to food, and we previously reported their impacts on subsequent reproduction (Grandmont et al. 2023; Legagneux et al. 2012). In this paper, we use additional physiological and demographic data from the same experiment, which was not included in prior analyses, to further investigate the impact of body condition and CORT stress‐response to cumulative stressors on survival and reproduction.

**FIGURE 1:**
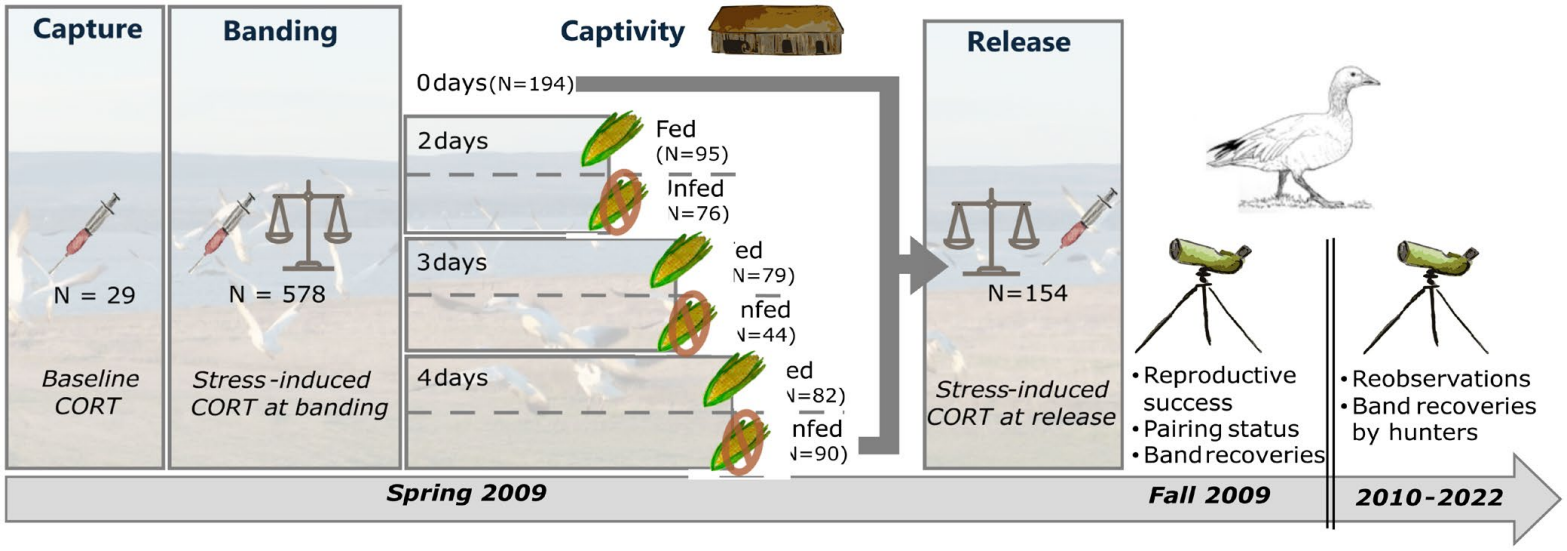
Graphical representation of the experimental design with the sequence of operations on greater snow geese. A maximum of three blood samples were taken for a given individual: within 3 min after capture to measure basal CORT level, at banding to measure stress‐response CORT and at release to measure prolonged stress response for experimental individuals. Control birds were released just after banding. Marked females were resighted in the field during the following autumn and the presence of young and mates were noted. Spring reobservations and recoveries of dead birds by hunters in subsequent years were used to model survival rates.

Body condition and stress level of staging geese were manipulated by capturing wild birds and maintaining individuals in captivity for 2–4 days, with or without access to food, before releasing them (Figure 1). Control individuals were released immediately after banding. Using physiological data (CORT levels and body condition), and reobservations and recoveries of marked individuals, we investigated potential effects of the experimental treatments and stress‐response on reproduction and survival in the following year. Specifically, we (i) quantified the relationship between experimental treatments and CORT levels; (ii) tested whether individual variations in CORT stress response could explain individual variations in demographic parameters in the year following the experiment; (iii) tested whether the accumulation of stressors from our experimental treatment groups affected survival the year following the experiment. We hypothesised that higher CORT levels would mediate the previously observed reduction in reproductive success of manipulated individuals (Grandmont et al. 2023; Legagneux et al. 2012). Considering that greater snow geese are long‐lived, we could expect major effects on reproduction but a negligible effect on survival, as the stressors were applied over a relatively short period, allowing for recovery to a sustainable allostatic load. Alternatively, we could also anticipate some reduction in survival due to the experimentally induced cumulative stress (LeTourneux et al. 2022).

## 2 Materials and Methods

### 2.1 Study Species and Study Site

The greater snow goose is a migratory species wintering along the Atlantic coast of the United States and breeding in the Canadian Arctic in Nunavut. This experiment took place in 2009 at l'Ile‐aux‐Oies (Québec; 47°00′ N 70°33′ W) in the St. Lawrence estuary, the major staging site used by geese from late March to late May to accumulate reserves for migration and reproduction (Gauthier et al. 1992). They also come back to this region during the autumn migration from the breeding grounds 3000 km further north. Geese have permanent pair bonds and stay in family units (i.e., young with their parents) for up to a year (Prevett and MacInnes 1980), so it is possible to quantify their reproductive success by determining the presence or absence of young with their parents from field observations during autumn staging in Québec. The proportion of young observed in the autumn flocks is a good proxy of reproductive success in this species and is highly variable from year to year, largely due to abiotic and biotic conditions encountered at the breeding site (Morrissette et al. 2010). The greater snow goose is a hunted species in autumn and spring in Quebec and in winter in the United States (LeTourneux et al. 2022). Adults can live on average for 10 years and up to 24 years (unpublished data). Their life history strategy is on the slow side of the fast‐slow continuum, meaning they tend to skip reproduction to optimise survival if their physiological condition and/or the environmental conditions are not favourable (Reed et al. 2004).

### 2.2 Capture, Measurements and Experimental Set‐Up

Geese were captured with cannon‐nets from late April to mid‐May 2009, mostly near the end of spring staging (Legagneux et al. 2012). As spring is the period of family break‐up (Gauthier and Tardif 1991), juveniles were released immediately after capture. The number of adults caught in each capture event ranged from 40 to 105. Adults were retrieved from under the nets and transported to a barn for banding and experimental manipulations (graphical representation in Figure 1). When groups were captured late in the day, they were banded the following morning, which was the case for 12 out of 22 captures. All adults were banded, sexed by cloacal examination, and females were measured (tarsus, culmen and head lengths to the nearest 0.1 mm), weighed (to the nearest gram) and blood samples were collected (see details below). Females were marked with yellow plastic neck collars to facilitate re‐observation at a distance after release.

Each capture group was assigned to a different treatment in a randomly predefined order. The control group was composed of individuals released immediately after banding, and treatment groups were composed of individuals subject to two different manipulations (see sample sizes in Figure 1): kept in captivity for 2, 3 or 4 days (captivity duration treatment) and either provided with food (crushed corn) or not (food treatment). Treatment groups were large and randomly selected, so any biases should be equally represented in all of them. Only females were involved in the experimental treatments because reproductive success is primarily related to female body condition (Bêty et al. 2003). Males were kept in captivity for the same amount of time as females, in separate indoor enclosures, with possible visual and vocal contact and with food. At release, females were weighed again, and blood samples were taken (see below). All females from the same capture group were assigned to a single treatment, and both males and females were released at once to prevent pair bond disruption (details in Legagneux et al. 2012). The experiment was shown to decrease body condition by 8% and 1.2% for unfed and fed groups, respectively (Legagneux et al. 2012). Also, time spent in captivity negatively affected reproductive success (Legagneux et al. 2012) through a reduction in breeding propensity (Grandmont et al. 2023).

Marked individuals were reobserved by our team and birdwatchers throughout the area used by staging geese along the St. Lawrence River every spring from 2009 to 2022. Individuals harvested by hunters in autumn, winter and spring in both Canada and the USA were also reported to us. This information enabled the estimation of survival with capture–mark–recapture models (LeTourneux et al. 2024). Collared females were also reobserved in autumn 2009 to record the presence of young and a partner with them, which we used as a measure of reproductive success and pairing status, respectively.

### 2.3 Blood Sample Collection and Analysis

Up to three different blood samples were taken from females at three different times: upon capture, during banding, and just before release. The first sample came from randomly chosen females sampled within 3 min at each capture, which reflects basal hormonal levels (Legagneux et al. 2011; Romero and Reed 2005). Only a small number of females could be sampled at each capture due to the 3‐min constraint. The second sampling was taken from all females (control and treatment groups) during banding (on average 11.0 ± [SD] 4.9 h after capture). Finally, the last blood sample was collected just before release from a random sample of females kept in captivity for 2, 3 or 4 days depending on the treatment group (Figure 1). Sample size for each group is detailed in Table S1. We used 23G needles and 1 mL syringes to collect 100 μL to 1 mL of blood from the tarsal vein. Blood samples were kept on ice for up to 4 h, then centrifuged at 8000 *g* for 8 min to separate the plasma from the red blood cells. These were then frozen separately at −20°C initially (max. 20 days) and at −80°C after field work. We dosed plasma samples (29 at capture, 578 at banding, 154 at release) for corticosterone concentration by radio‐immuno‐assays as described in Lormée et al. (2003) at the Centre d'Etudes Biologiques de Chizé (France). Two assays were performed for each sample, and the intra‐assay coefficient of variation was 2.17% (*N* = 104).

### 2.4 Statistical Analyses

To obtain an index of endogenous reserves independent of individual size, we corrected the body mass of females by skeletal measurements. We ran a principal component analysis (PCA) on tarsus and culmen lengths, using individuals captured and measured at the same site from 2006 to 2009. We used the first component (PC1), explaining 71% of the variance, in a linear regression with body mass as the dependent variable and added the residuals of this model to the mean mass. We then added the residuals of this linear model to the mean mass corrected by size to correct for the date of capture (see LeTourneux et al. 2021 for details). We thus obtained an index of fat reserves independent of skeletal size and capture date, which we will henceforth refer to as ‘body condition’.

Because of the high variability in time elapsed between capture and blood sampling at banding, we tested for an effect of this variable on CORT levels. We used a linear model as the quadratic model was not preferred and less parsimonious (ΔAIC = 1.33 in favour of the linear regression). We found a significant negative relationship between CORT levels at banding and time between capture and sampling (*β* = −0.01, 95% CI = [−0.03, −2.41e‐03]; *N* = 578; Figure S2). We thus used the residuals of the previous model as the CORT levels at banding corrected for the influence of time elapsed since capture in subsequent analyses, similarly to our approach for body condition. No such relationship was found in individuals measured at release (*β* = −4.98e‐0.4 [−0.01, 4.92e‐03]; *N* = 154), possibly due to a smaller number of individuals processed. Nonetheless, we applied the same correction for samples collected at release as for those collected at banding, for consistency. However, this correction did not apply to samples at capture because they were all taken in less than 3 min and the exact time needed to take the sample was not recorded.

Because geese are fattening during spring staging and thus their physiological state may change over time, we also tested for an influence of capture date on all three types of blood samples. We found a significant relationship between CORT and capture date in samples at capture and at banding (*β*_capture_ = −1.48 [−2.84, −0.11], *N* = 29; *β*_banding_ = −1.42 [−2.00, −0.84], *N* = 560; *β*_release_ = −0.69 [−2.03, 0.64], *N* = 154; Figure S3). We used the residuals of these models to correct for capture date in all sample types, again for consistency. Corrected CORT values were the residuals of the regression added to the mean CORT value for each sample type and these values were used for all following analyses.

We tested for a difference in CORT concentration between the three sample types (capture, banding and release) using a two‐way mixed‐effect ANOVA with individual as a random effect to account for measures on the same individuals (lmer function, lme4 package, Bates et al. 2024). A significant difference was found (see Section 3), and thus we used CORT concentrations during banding corrected by time since capture and date of capture (referred to as stress‐induced CORT at banding) as an indicator of the intensity of the stress response in subsequent analyses.

We tested the repeatability of the stress‐induced CORT concentration at banding on nine individuals caught and sampled at two distinct capture events during the staging period. Repeatability analyses were conducted using the intraclass correlation coefficient (ICC), which uses a one‐way ANOVA to estimate intra‐ and inter‐group variance (Wolak et al. 2012) (‘single_fixed_raters’ index in ‘ICC’ function from ‘psych’ R package, Revelle 2024) and a Spearman correlation. Those nine recaptured individuals were removed from all subsequent analyses (*N* = 560 hereafter).

We tested for differences in stress‐induced CORT levels at banding (i.e., before the start of the experiments) between treatment groups using an ANOVA and a Tukey post hoc test. Relationships between stress‐induced CORT levels at banding or at release and food treatment, number of days spent in captivity, and body condition were investigated using linear models. Normality and homoscedasticity of the residuals were graphically confirmed for these linear models. We also examined the influence of stress‐induced CORT at banding on reproductive success (presence vs. absence of young in autumn) using generalised linear models of the quasibinomial family. We included an interaction with time spent in captivity (random effects impossible with quasibinomial family) to control for the known effect of captivity on reproductive success (Grandmont et al. 2023). In all statistical analyses, we selected the best model explaining variations in our response variable using AICc, and we used 95% confidence intervals (reported throughout) around the beta estimates to evaluate significance.

We used a multi‐event, capture‐mark‐recapture model (Pradel 2005) combining live reobservations with dead recoveries (bands reported by hunters) to analyse survival of collared females, while controlling for probability of encounter. Marking occurred in a single year (spring 2009), but reencounters extended over a 13‐year period. Extending the period to collect reencounters increased the precision of the estimation of survival in the first year following the experiment, which was of primary interest. On each occasion, an individual could be observed alive (coded 1), recovered (2) or not encountered (0). Individuals recovered dead between spring *t* and *t* + 1 were coded dead at spring *t* + 1 (Gauthier and Lebreton 2008). The model is a simplification of LeTourneux et al. (2022) and could estimate survival (*S*), live‐encounter (*p*) and recovery (Seber's *r*) probabilities. We examined the influence of stress‐induced CORT level at banding, pre‐ and post‐treatment body condition, food treatment (fed vs. unfed during captivity) and number of days spent in captivity (0, 2, 3 or 4 days) on survival in the first year after release. Continuous variables like CORT levels and body condition were standardised. A maximum two covariates were included in each model because of restrictions due to sample size. We did not include pre‐ and post‐treatment body condition, or food treatment and post‐treatment body condition in the same models because these variables were highly correlated. We conducted model selection sequentially, first on recovery, then on reobservation and finally on survival probabilities using QAICc. After a first selection of the best survival models, we tested again for more complex models on reobservation and recovery parameters. Models with a difference in QAICc of 2 or less from the best model were considered competitive. The capture‐recapture analysis was conducted with program E‐SURGE V.2.2.3 (Choquet, Rouan, and Pradel 2009). Prior to conducting the survival analysis, we tested the goodness‐of‐fit of the general model with the program U‐CARE V2.3.5 (Choquet, Lebreton, et al. 2009). We found a *χ* ^2^ value of 71.4 for 63° of freedom, which yielded an overdispersion coefficient $\left(\hat{c}\right)$ of 1.134. We used this value in the capture–recapture analysis, which explains our use of QAICc rather than AICc. Because we found that the food treatment affected survival (see Section 3), we compared a posteriori the raw hunter recovery probabilities of fed, unfed and control in the first year after the experiment using a binomial linear model with a logit link.

## 3 Results

### 3.1 Stress Response and Factors Affecting CORT Levels

A total of 560 adult females, excluding females sampled twice, were captured, banded, and released in this study. Among them, 184 were control birds released immediately after banding, and 377 birds were kept in captivity for 2–4 days with or without access to food (203 and 174 individuals respectively).

**FIGURE 2:**
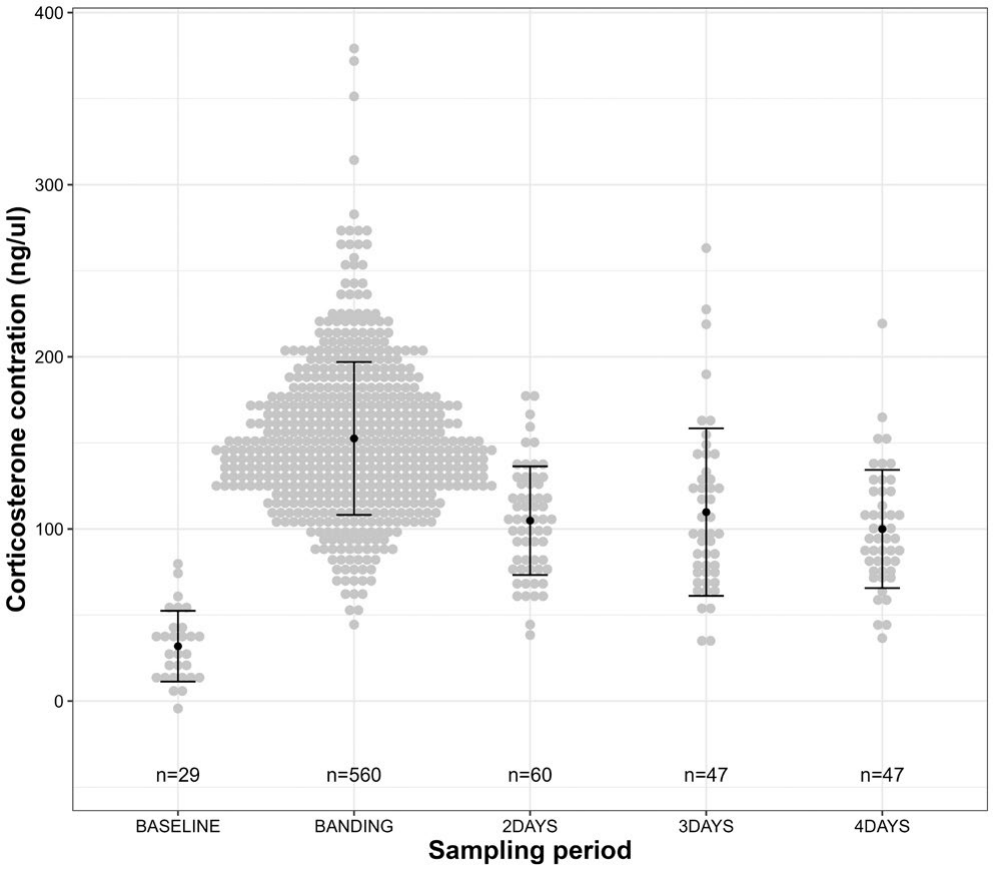
Corticosterone level of greater snow geese measured within 3 min of capture (baseline level), at banding (11.0 ± [SD] 4.9 h after capture) and at release (2, 3 or 4 days after capture). Individual data points are represented in grey and mean and standard deviation in black. The CORT values are corrected for time before blood sampling and capture date. Individuals sampled twice were removed.

CORT concentrations differed depending on when blood sampling occurred (Figure 2). Baseline level taken within 3 min of capture (31.88 ± [SD] 20.57 ng/μL; *N* = 29) was much lower than stress‐induced level at banding measured 11 h after capture on average (152.56 ± 44.43 ng/μL; *N* = 560; *β*_Banding/Baseline_ = 117.23 [101.75, 132.76]). The stress‐induced CORT level decreased after 2–4 days spent in captivity (104.85 ± 38.28 ng/μL; *N* = 154; *β*_Release/Banding_ = −50.72 [−58.06, −43.28]) but remained higher than baseline level (*β*_Release/Baseline_ = 66.50 [49.80, 83.34]) and did not vary between 2 and 4 days of captivity (*β*_Days in captivity_ = −2.15 [−9.51, 5.22], Figure 2). Stress‐induced CORT levels at banding (before the experiment) were similar between individuals selected for different food and capture duration treatments (see Table S4). Intra‐individual baseline and stress‐induced CORT levels at banding were unrelated (*β* = 0.44 [−6.58, 7.45]; *N* = 29).

**FIGURE 3:**
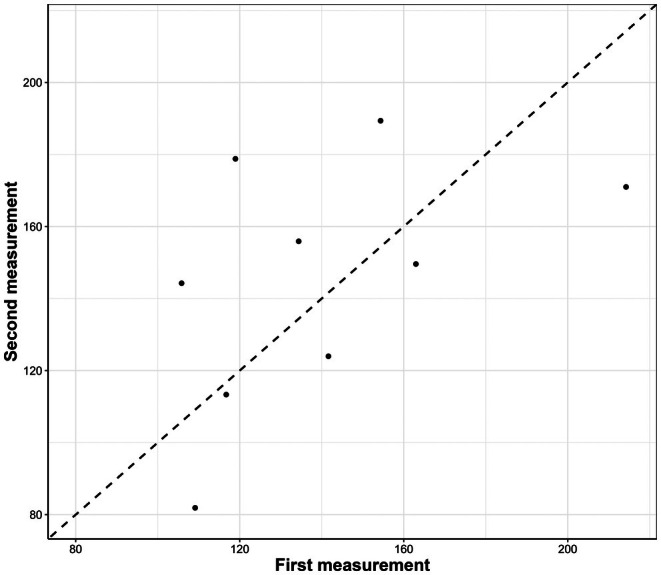
Stress‐induced corticosterone levels at banding (corrected by the time before sampling and capture date), for individuals with repeated measures. Dashed line represents 1:1 ratio for reference.

Repeatability of stress‐induced CORT levels at banding for the nine individuals captured twice was relatively high but non‐significant (ICC = 0.52 [−0.1, 0.9]; *ρ* = 0.55; *p* = 0.13; Figure 3).

**FIGURE 4:**
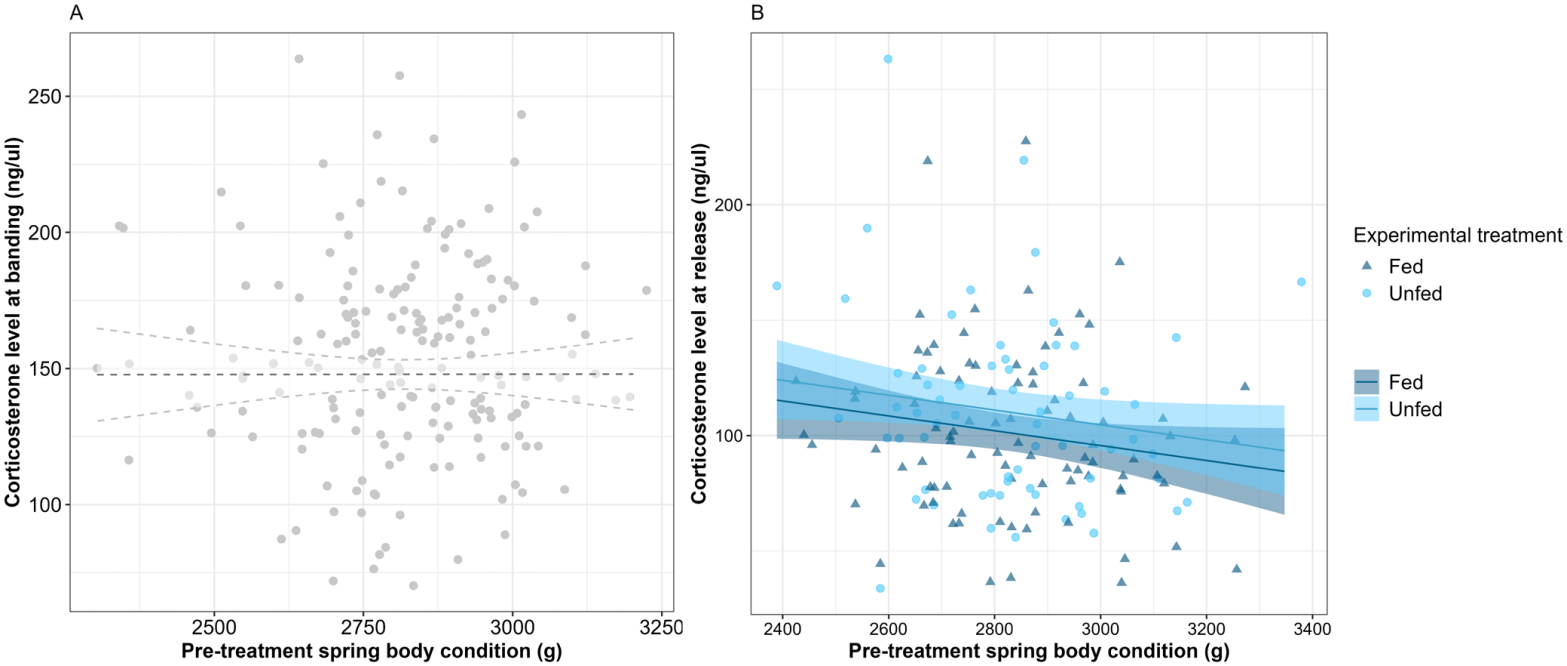
Relationship between corticosterone level (corrected by the time before sampling and capture date) of greater snow geese at banding (A) or at release (B) and pre‐treatment spring body condition (mass at banding corrected by size and date of capture). Mean predictions and 95% confidence intervals are presented [from model MC1, refer Table 1, for (B)].

We found no significant relationship between stress‐induced CORT level at banding and pre‐treatment spring body condition (*β* = −0.04 [−5.32, 5.40]; Figure 4A) or post‐treatment body condition (*β* = −0.02 [−0.04, 7.42e‐03]). The stress‐induced CORT level at release was marginally inversely related to pre‐treatment spring body condition (*β* = −5.96 [−12.02, 0.10]; Figure 4B, MC2 in Table 1), and tended to be higher in the unfed than the fed group (*β*_fed/unfed_ = 9.19 [−3.13, 21.51]; Figure 4B, estimates from model MC3 in Table 1) but was not related to the number of days spent in captivity (*β*_Day3/Day2_ = 4.97 [−9.79, 19.72]; *β*_Day4/Day2_ = −4.85 [−19.60, 9.91]).

**TABLE 1:** Selection of linear models examining the influence of food treatment (fed or unfed), pre‐treatment spring body condition (mass at banding corrected for size) and days spent in captivity (2, 3 or 4 days) on CORT level of greater snow geese at release.

Model names	Factor included	*K*	Log likelihood	Delta AICc
MC1	Condition + food	4	−776.38	0.00
MC2	condition	3	−777.44	0.02
MC3	Food	3	−778.24	1.62
MC.NULL	Intercept	2	−779.33	1.72
MC4	Food × condition	5	−776.30	1.98

*Note:* Only models with a AICc lower than the null model are reported here.

### 3.2 Influence of Stress Response on Fitness Parameters

Our proxy for reproductive success (presence of young with females in autumn) was not related to stress‐induced CORT at banding or at release (*β*_CORTbanding_ = −7.72e‐03 [−0.02, 7.50e‐03] and *β*_CORTrelease_ = −0.03 [−0.11, 0.04]).

In our survival analysis, preferred models retained a yearly variation in recapture but constant recovery rates (see Table S5 for full details). Our top four models were all within two points of QAICc, suggesting a strong influence of the food treatment and a weaker influence of pre‐treatment spring body condition and stress‐induced CORT level at banding on survival during the first year after release (Table 2). However, there was no evidence for an influence of the number of days in captivity on survival (Table S5) and the survival rate was constant over time after the first year.

**TABLE 2:** Results of model selection analyzing survival of greater snow geese marked in spring 2009 in relation to several covariates.

Model name	First year survival	Recapture	Recoveries	*K*	Deviance	ΔQAICc
MS24	FOOD	*t*	Constant	17	2845.63	0
MS27	FOOD + COND1	*t*	Constant	18	2844.50	1.08
MS5	FOOD	Constant	Constant	6	2872.35	1.10
MS30	CORT + FOOD	*t*	Constant	18	2845.18	1.67
MS6	FOOD + COND1	Constant	Constant	7	2871.17	2.09
MS7	CORT + FOOD	Constant	Constant	7	2871.85	2.69
MS35	COND2	*t*	Constant	14	2850.98	2.77
MS36	Null	*t*	Constant	14	2855.77	2.77
MS8	COND2	Constant	Constant	5	2877.85	3.9

*Note:* Covariates apply only to survival in the first year after release and include stress‐induced corticosterone level at banding (CORT), spring body condition (COND1: Condition at banding; COND2: Condition at release), food treatment (FOOD: Treatment fed/unfed/control), and number of days spent in captivity (Days: 0, 2, 3 and 4 days). Time (*t*, in years) was tested only on recovery and recapture probabilities. Survival was constant after the first year in all models. *K*, number of parameters; ΔQAICc, difference in QAICc between the current and the top‐ranked model. See Table S5 for full model selection.

**FIGURE 5:**
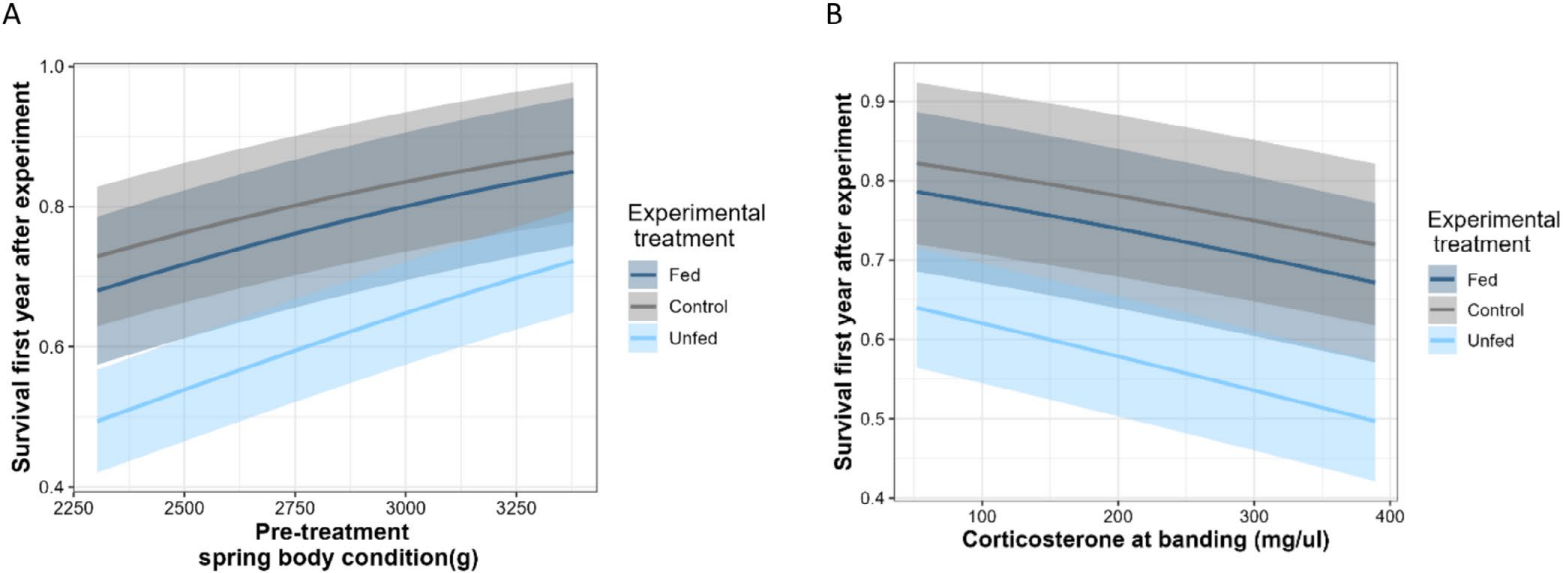
Relationship between survival of greater snow geese in the first year after the experiment and pre‐treatment spring body condition (A) or stress‐induced corticosterone level (B) for birds kept in captivity with or without food for up to 4 days or released immediately after banding (control). Mean survival predictions with 95% confidence intervals are from parameters of models MS31 (A) and MS34 (B) in Table 2.

Survival in the first year after release was similar in control and fed birds but lower in unfed birds (effect of food treatments compared to the control group: *β*_fed_ = −0.24 [−1.06, 0.57]; *β*_unfed_ = −0.98 [−1.75, −0.21]; Model MS24 in Table 2; Figure 5). Survival tended to be positively related to pre‐treatment spring body condition (*β*_cond1_ = 0.16 [−0.16, 0.49]; Model MS27 in Table 2; Figure 5A), and negatively related to stress‐induced CORT level at banding (*β*_cort_ = −0.09 [−0.36, 0.19]; Model MS30 in Table 2; Figure 5B). Although the 95% confidence intervals included zero, models with pre‐treatment spring body condition and stress‐induced CORT level at banding were retained as competitive models (Table 2).

**FIGURE 6:**
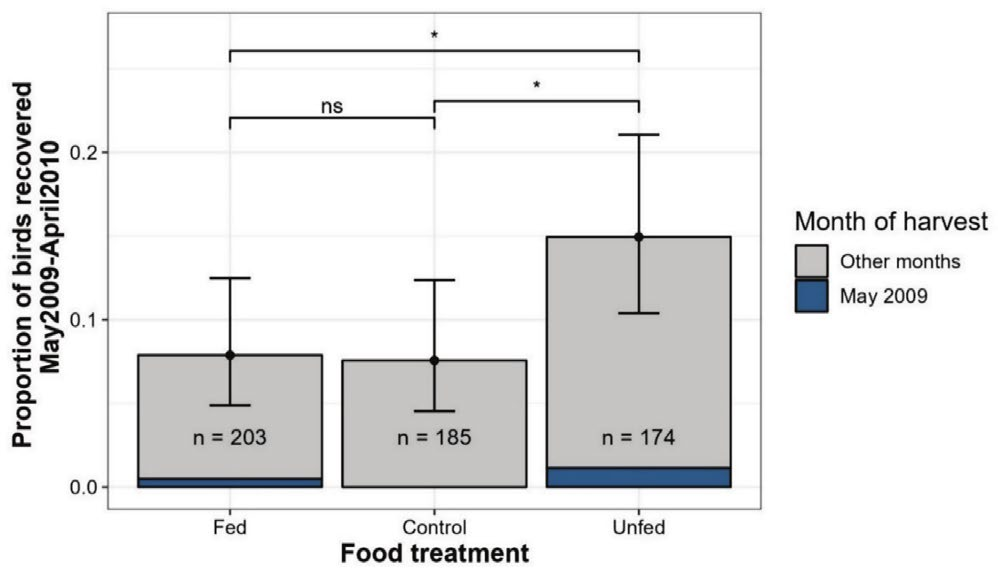
Recoveries of banded greater snow geese in the first year post‐release as a function of the food treatment groups. Grey bars are the proportions from the raw data, the black points and error bars are the mean predictions and 95% confidence intervals from a binomial generalised linear model. * means the difference is significant. ns means the difference is non significant.

When examining band recoveries between the food treatment groups, we found that a higher proportion of birds from the unfed treatment were recovered by hunters in the first year after release compared to birds in the control group (*β*_unfed/control_ = 0.75 [0.07, 1.46]) or compared to the fed group (*β*_unfed/fed_ = 0.78 [0.12, 1.48]; Figure 6). On the contrary, fed individuals were harvested at a similar rate (*β*_fed/control_ = −0.04 [−0.80, 0.73]; Figure 6).

## 4 Discussion

Our study experimentally simulated an unpredictable stressful event during the spring staging of a long‐distance migratory bird species to examine the consequences of the physiological response to multiple stressors and of a body condition reduction on reproductive success and survival. Our large sample size allowed us to describe the stress response of cumulative stressors and its consequences on survival despite the confounding environmental factors associated with any field experiment. The combination of physiological parameters and capture‐recapture data enabled us to partition the effects of individual variation in body condition and intensity of stress response. Contrary to our first hypothesis, we found that the hormonal stress response did not explain individual variation in reproductive success. However, all geese exhibited a prolonged stress response during the 2–4 days spent in captivity, which likely explains the effect of captivity on breeding suppression found in a previous study (Grandmont et al. 2023). We found evidence for carry‐over effects of the experiment on survival in the following year as it was reduced for unfed individuals, which cumulated the most stressors, thus supporting our alternative hypothesis regarding survival.

### 4.1 Proxy of Stress Response and Individual Variation

We measured three distinct CORT levels throughout our experiment: at capture (baseline), at banding (on average 11 h after capture, stress‐induced) and at release from captivity (2–4 days later, also stress‐induced). The maximal stress response was observed at banding, and this response appeared repeatable in individuals recaptured later in the same season. Though our repeatability analysis was non‐significant, our effect size was higher than what is usually reported for stress‐induced CORT levels (0.52 vs. 0.38 according to a meta‐analysis by Taff et al. 2018). Considering our small sample size, the non‐significance could be caused by a lack of statistical power, not by an absence of repeatability. Individuals may respond differently to the same situation, maintaining a diversity of coping styles within a given population (Cockrem 2013; Taff et al. 2018), which is associated with distinct energy allocation strategies (Angelier et al. 2018). This heterogeneity in CORT response has been proposed as a mechanism to help explain individual heterogeneity in performance (Angelier et al. 2018; Cockrem 2013; Wada et al. 2008). Interestingly, stress‐induced CORT levels were found to be more repeatable than baseline CORT levels in the meta‐analysis of Taff et al. (2018). This may suggest that individual variation in CORT response reflects a personality‐like trait, potentially indicating differing sensitivities to multiple environmental stressors. This result supports our exploration of the role of stress‐induced CORT levels at banding in modulating reproduction and survival.

Most studies with comparable experimental designs have been conducted on other bird taxa. Nonetheless, experiments on wild passerines captured and kept in captivity have shown an immediate CORT increase in individuals deprived of food, but CORT levels of fed and unfed individuals were similar 1 day after the beginning of fasting (Lynn et al. 2003). In wild seabirds, naturally occurring food deprivation increased circulating CORT levels of individuals (Angelier et al. 2015; Kitaysky et al. 2007). After prolonged captivity, fasting and non‐fasting individuals decreased their circulating CORT levels similarly (Angelier et al. 2015). Because our experimental design was built to minimally disturb the geese during captivity, we were not able to look for an initial increase of CORT immediately after the start of the food restriction for unfed individuals or to compare the pattern of CORT decrease between treatment groups. Stress‐induced CORT levels at release tended to be higher for unfed than for fed individuals, which is coherent with the idea of an allostatic overload as the energy necessary to respond to the stress of captivity exceeded the energy available (McEwen and Wingfield 2003). Moreover, the stress‐induced CORT levels at release were higher than baseline values but lower than the peak response measured at banding, which is typical of the downregulation that occurs during chronic stress (Rich and Romero 2005; Romero 2004). Although the duration of our experiment is too short to fit the formal description of chronic stress (Romero 2004), we believe that similar mechanisms occurred in our experiment. In fact, geese in our experiment were not habituated because they were not repeatedly handled during captivity, nor exhausted because they were able to fly away immediately after release, suggesting they downregulated their CORT response as in Rich and Romero (2005). Under acute stress conditions, the stress response and its associated energy allocation pathways are saturated, making individuals unable to respond to a new stressor with the same intensity (Romero 2004). This mechanism has also been observed in other bird taxa such as passerines (Angelier et al. 2015; Cockrem 2013).

Interestingly, we had some evidence that stress‐induced CORT levels at release could be modulated by pre‐treatment spring body condition. This could indicate that individuals in better condition, with higher endogenous reserves, presented a lower stress response than those in lower condition, even under prolonged stressors. A similar relationship was found after reproduction in Adelie penguins ( *Pygoscelis adeliae* ; Cockrem et al. 2006). This suggests that variations in CORT levels after an acute or prolonged stressor can be modulated by the individual state and the amount of body reserves.

### 4.2 Carry‐Over Effects of Multiple Stressors on Fitness Parameters

We investigated the role of stress‐induced CORT level on fitness parameters, which has been less studied than the links with baseline CORT level (Sorenson et al. 2017; Bonier et al. 2009; Breuner et al. 2008). Results reported in the literature highly depend on the fitness trait being studied but seem to show a stronger association of baseline levels with reproductive success than with other demographic traits, especially in long‐lived seabird species (Sorenson et al. 2017).

In our study, variations in stress‐induced CORT levels, assumed to be an index of the intensity of the hormonal stress response, did not explain differences in reproductive success. On the other hand, Legagneux et al. (2012) and Grandmont et al. (2023) found that time spent in captivity was the main driver of a reduction in reproductive success in our experiment. The effect was stronger in 2009, when conditions were unfavourable for migration and breeding, offering fewer opportunities to recover from stress experienced during the spring (Grandmont et al. 2023; Legagneux et al. 2012). This was also the year we collected blood samples. We thus cannot exclude that covariations between CORT response and reproductive success might have occurred in years with better environmental conditions. However, a longer time spent in captivity will translate into more time spent in the state of hormonal saturation, as described above, and we suggest that it may explain the decrease in breeding propensity reported in Grandmont et al. (2023).

The effects of stress‐induced or acute GC levels on survival are not fully understood, nor are the patterns clear (Schoenle et al. 2018; Breuner et al. 2008) as it is generally context‐dependent (Crespi et al. 2013; Bókony et al. 2009; Cockrem et al. 2006). For example, a positive relationship between survival and CORT levels was found in poor‐quality habitats but not in high‐quality habitats for a passerine species (Angelier et al. 2009) and an inverse tendency was shown in a group of long‐lived species (Schoenle et al. 2021). In this context, our results provide evidence that stress‐induced CORT alone does not explain variations in survival but may reduce survival in addition to food deprivation, which supports the results of LeTourneux et al. (2022) regarding the impact of cumulated stress on survival. Unfed individuals lost up to 8% of their endogenous reserves during captivity (Legagneux et al. 2012) and were less likely to survive the following year compared to controls and fed individuals. Reduced energy reserves and the accumulation of stressors during spring staging therefore appear to be potential predictors of subsequent survival. Considering that baseline and stress‐induced CORT were not predictive of one another, we also suggest that they have complementary roles in the regulation of fitness traits, as previously suggested (Angelier and Wingfield 2013; Landys et al. 2006; Romero 2004).

It is important to note that all captured females (experimental and control groups) were equipped with plastic collars, which were shown to interact with hunting in reducing survival, likely by inducing a negative effect on body condition (LeTourneux et al. 2022). As stated earlier, 2009 had rather unfavourable environmental conditions. It is thus possible that under better conditions, the stress‐induced effects that we observe here might have been mitigated (Grandmont et al. 2023). On the other hand, we can argue that these unfavourable environmental conditions were a supplementary stressor on top of those imposed by our experimental conditions. As a result, geese had to face the cumulated stress generated by capture, time spent in captivity, food restriction, wearing a neck collar, and unfavourable environmental conditions in 2009. This could heighten the impact on survival, as multiple stressors can have consequences more severe than the sum of each due to synergistic effects (LeTourneux et al. 2022). Nonetheless, the differences found between the food treatment groups can only be attributed to this treatment because all individuals were equipped with the same collars. Similarly, pre‐experiment body condition, baseline, and stress‐induced CORT levels cannot be affected by the collar for the same reason, so our interpretations of the links between these physiological parameters and survival remain valid.

Hunting is a major source of mortality in our population (LeTourneux et al. 2022), potentially affecting individuals differently depending on their individual allostatic state (Dehorter and Tamisier 1998). We consequently looked at recovery rates in the year following the experiment, which can be an index of hunting mortality. This could provide insights into the possible role of this factor in the differences in survival found between our experimental groups. Recovery rates by hunter were higher for unfed individuals than for control and fed individuals, and most mortalities occurred in subsequent autumn and winter, not immediately after release. This suggests a carry‐over effect of the food treatment on survival, at least partly through hunting mortality. Captivity but not food treatment was shown to decrease reproductive success (Grandmont et al. 2023; Legagneux et al. 2012), so fed and unfed individuals had comparably low reproductive success. Therefore, it is unlikely that differences in hunting vulnerability related to reproductive success (e.g., due the presence of naive juveniles accompanying the parents, Calvert and Gauthier 2005) can explain the high hunting mortality of the unfed group. We offer three mutually non‐exclusive hypotheses to explain our result.

1. *The body condition hypothesis*: Geese exposed to prolonged stressors may decrease their foraging behaviour for an extended period of time after the event. The reduction in body condition incurred might thus still be present in autumn, forcing individuals to forage in higher quality but riskier habitats, namely crop lands (LeTourneux et al. 2021), where most of the hunting activity takes place. A prolonged reduction in body condition also underlies the other hypotheses through different mechanisms.
2. *The moult hypothesis*: A reduced body condition could hinder moulting, producing lower quality feathers. Since geese moult their flight feathers only once a year during the summer (Marmillot et al. 2016), low quality flight feathers can have long‐lasting carry‐over effects by increasing allostatic load, thereby impacting body condition and survival (Johns et al. 2018). This could translate into reduced abilities to escape from hunters or increased energy costs for flight, generating more risk‐taking behaviours to forage (see Hypothesis 1), thus increasing vulnerability to hunting.
3. *The pair‐bond disruption hypothesis*: Chronic stress imposed by marking with radio‐transmitters was shown to increase pair‐bond disruption by 25% (Demers et al. 2003). Although males and females were released at the same time to avoid pair break up, a change in behaviour or reduced body condition in unfed females after release could have increased the pair‐bond disruption rate in this group (Choudhury 1995), which could be supported by the decrease in breeding propensity found in Grandmont et al. (2023). Unpaired geese have a subordinate status in flocks (Stahl et al. 2001), leading to lower body condition, increased vulnerability to hunters (see Hypothesis 1) and reduced survival (Leach et al. 2020). We were able to indirectly test this hypothesis using resightings of birds with or without a mate during autumn 2009 in Québec (Legagneux et al. 2012). In a posteriori analysis (Figure S6), we found evidence for a negative effect of days spent in captivity on the pairing status in autumn (Figure S6.1A) but not of the food treatment (Figure S6.1B). It was not possible to directly test for the effect of pairing status on survival because of low sample size, but by using the probability of band recovery, we found that unpaired birds, especially those in low post‐treatment body condition, had a higher recovery rate than paired ones (Figure S6.1C,D). Although these analyses offer ambiguous support for this hypothesis, investigation of the overall effect of pair‐bond disruption on vital rates appears promising for future work.

Even if we cannot conclude with certainty which mechanisms could be the main drivers of the observed carry‐over effect on survival, a sustained lower body condition, possibly mediated by reduced‐quality feathers and pair‐bond disruption, could explain the prolonged effects of cumulative stressors lasting up to 10 months after the experiment.

## 5 Conclusion

Our experiment simulated unpredictable and cumulative stress conditions during an important energy demanding period of the life cycle. Cumulative stressors can lead to an allostatic overload that results not only from the hormonal reactivity of individuals but also from the limited energy available that can either be allocated to maintenance or reproduction (Romero et al. 2009; McEwen and Wingfield 2003). This response to stressors is modulated by individual heterogeneity in the initial energy state (i.e., pre‐treatment spring body condition) and the intensity of the hormonal response (Cockrem 2013). Manipulating body condition provided evidence that individual state is key to coping with unpredictable stressors. This was found in a partial capital breeder that can accumulate substantial endogenous reserves to adjust to environmental stochasticity (Gauthier et al. 2003). We could thus expect an even stronger response in species that cannot accumulate large body reserves. The carry‐over effects observed several months after imposing an unpredictable stress also highlight how long‐lived individuals can adjust life history decisions (e.g., by reducing reproductive investment) based on their state, which can take a long time to recover from some perturbations. However, when stressors were accumulated (e.g., kept in captivity without food after a stressful capture event), individuals were less able to cope with the increased allostatic load (Romero et al. 2009; McEwen and Wingfield 2003), which ultimately reduced survival. Even if the pair‐bond disruption hypothesis remains a possible mechanism to explain our results, further studies should be dedicated to quantifying more directly the effect of pair‐bond disruption on survival in long‐lived monogamous species. Experimental designs in wild populations are difficult due to the many potentially confounding factors involved, necessitating large sample sizes to compensate for them, which can explain their low occurrence in the literature. Nonetheless, experimental studies such as ours remain a promising avenue to understand the mechanisms linking physiology and fitness to multiple environmental stressors that can interact with each other (Crespi et al. 2013).

## Author Contributions

**Ilona P. Grentzmann:** data curation (equal), formal analysis (lead), investigation (equal), validation (equal), visualization (lead), writing – original draft (lead), writing – review and editing (lead). **Gilles Gauthier:** conceptualization (supporting), formal analysis (supporting), funding acquisition (equal), investigation (supporting), project administration (supporting), resources (lead), supervision (supporting), validation (supporting), visualization (supporting), writing – original draft (supporting), writing – review and editing (supporting). **Frédéric Angelier:** formal analysis (supporting), methodology (supporting), writing – review and editing (supporting). **Joël Bêty:** conceptualization (equal), formal analysis (supporting), funding acquisition (equal), investigation (equal), methodology (equal), project administration (equal), resources (supporting), supervision (supporting), validation (supporting), writing – original draft (supporting), writing – review and editing (supporting). **Frédéric LeTourneux:** formal analysis (supporting), supervision (supporting), validation (supporting), visualization (supporting), writing – original draft (supporting), writing – review and editing (supporting). **Pierre Legagneux:** conceptualization (equal), data curation (equal), formal analysis (supporting), funding acquisition (equal), investigation (equal), methodology (equal), project administration (equal), resources (supporting), supervision (lead), validation (lead), visualization (supporting), writing – original draft (supporting), writing – review and editing (supporting).

## Ethics Statement

This experiment was approved by the Committee of Animal Protection of the Université du Québec à Rimouski (Authorization number: CPA‐42‐10‐78).

## Conflicts of Interest

The authors declare no conflicts of interest.

## Supporting information


Data S1.

## Data Availability

All data and codes are available from the Dryad Digital Repository: https://doi.org/10.5061/dryad.c2fqz61jr (Grentzmann et al. 2025).
