# Supplementary material for: Manipulating Individual State During Migration: Carry‐Over Effects of Cumulative Stress on Survival
Source: Ecol Evol. 2025 Jul 27;15(7):e71812. doi: 10.1002/ece3.71812 (PMC12300472; doi:10.1002/ece3.71812)
Supplement: Supplementary file 1 — Data S1. [file ECE3-15-e71812-s001.docx]

**Manipulating individual state during migration: carry-over effects of cumulative stress on survival - Supplementary materials**

*Table S1: Details of the number of samples and observations collected for our analyses*

| **Data or sample type** | **Number of samples or observations** |
| --- | --- |
| Baseline CORT | 29 |
| Stress-induced CORT at banding | 578 |
| Stress-induced CORT at release | 154 |
| Body condition at banding | 560 |
| Body condition at release | 154 |
| Total number of birds recovered in 2009-2010 | 56 |
| Total number of birds reobserved in autumn 2009 | 37 unpaired; 131 paired |

| 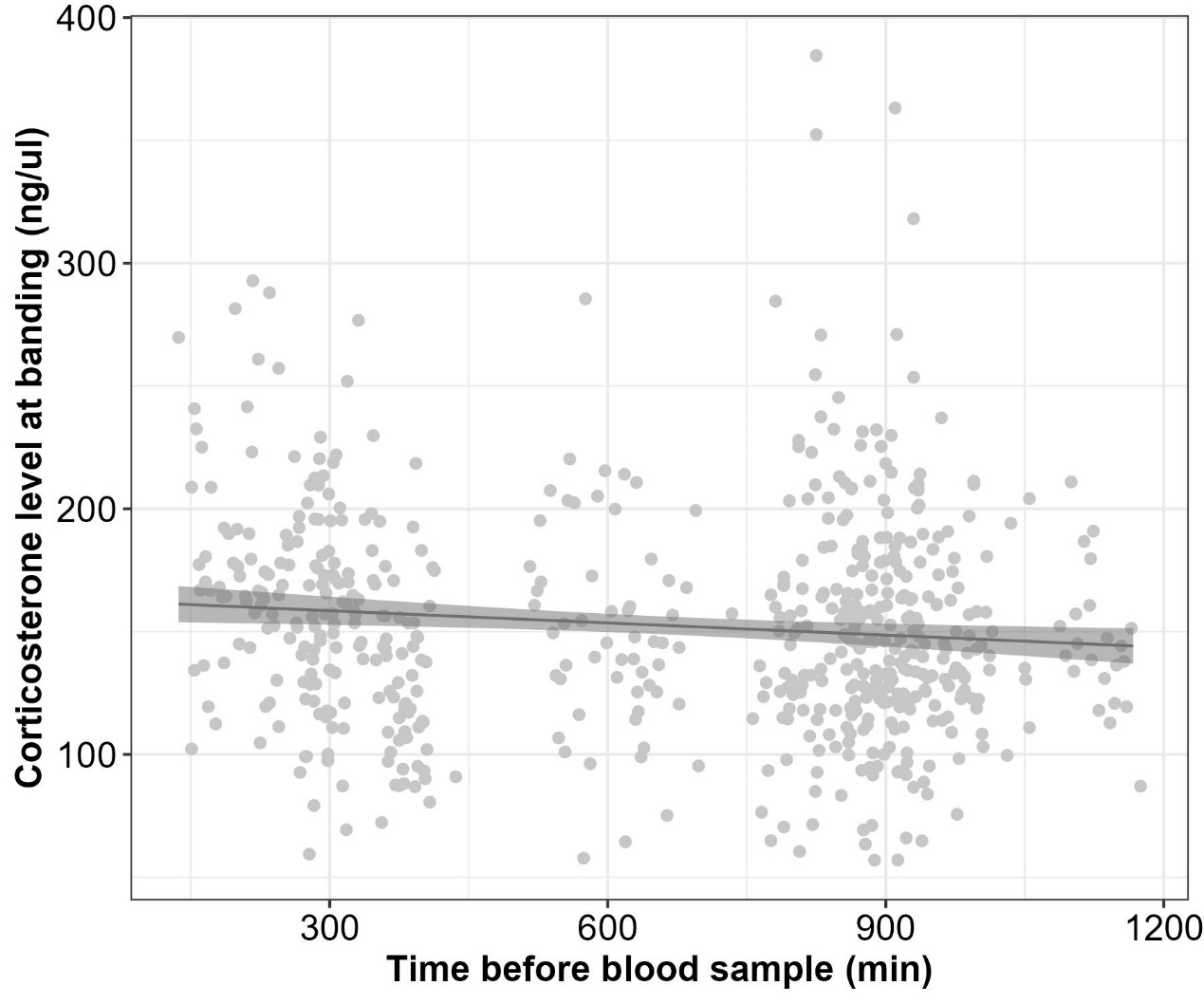 |
| --- |
| *Figure S2: Relationship between corticosterone level measured at banding (stress-induced CORT) and time elapsed between capture and blood sampling during banding. Regression with and 95% confidence intervals (shading).* |


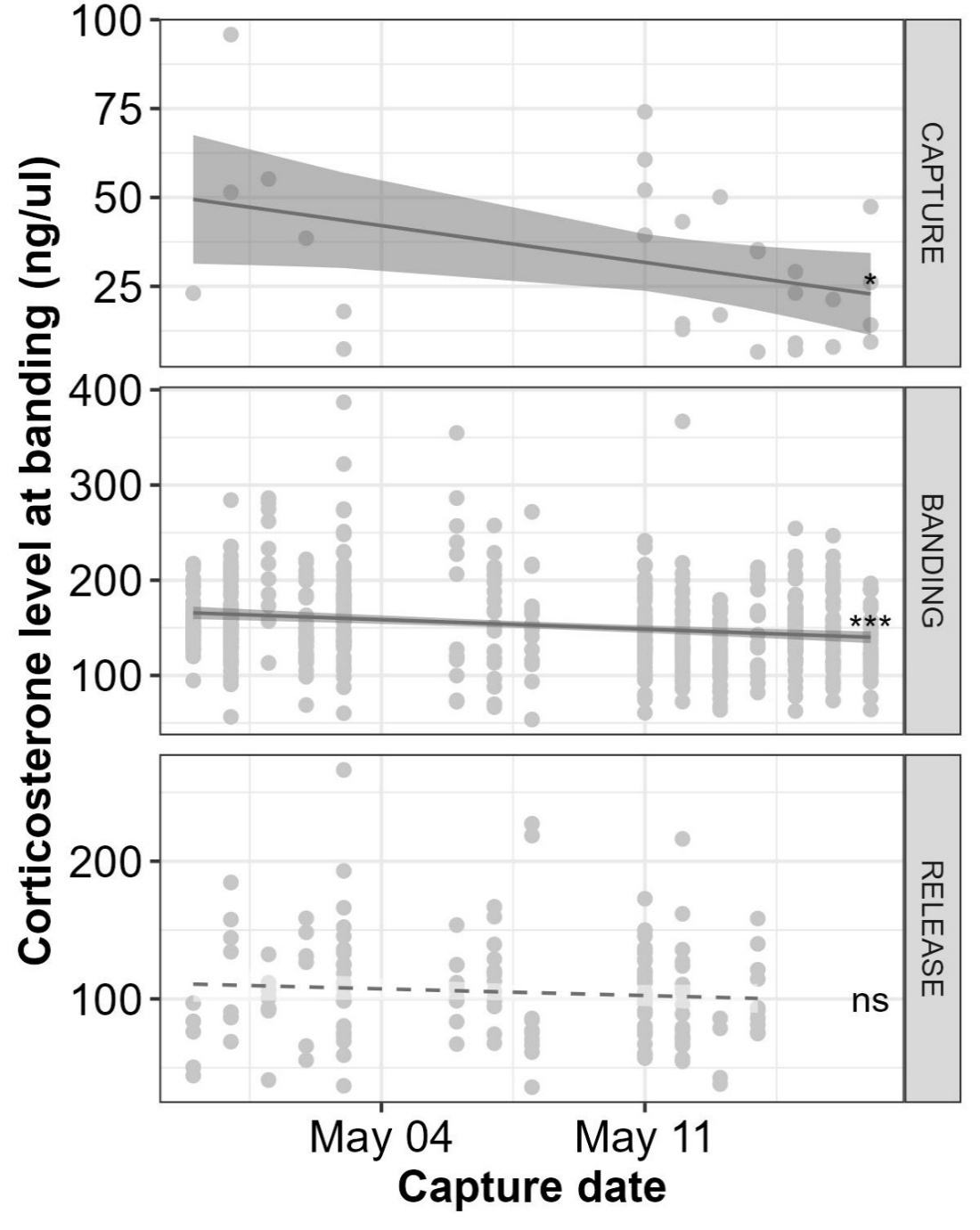


*Figure S3: Relationship between corticosterone level and capture date for each sample type. Regression with and 95% confidence intervals (shading) when the relationship was significant.*

*Table S4: Validation that CORT levels were similar between individuals before they were randomly assigned to treatment groups. Estimates and 95% confidence intervals from TukeyHSD post-hoc tests on ANOVAs.*

| **Two by two comparisons** | **Beta** |
| --- | --- |
| Fed-Control | -3.14 [-13.80; 7.52] |
| Unfed - Control | -0.38 [-11.43; 10.68] |
| Unfed - Fed | 2.76 [-8.05; 13.57] |
| 2 days - Control | -2.75 [-15.63; 10.14] |
| 3 days - Control | 2.16 [-11.91; 16.22] |
| 4 days - Control | -4.07 [-17.12; 8.97] |
| 3 days - 2 days | 4.90 [-9.96; 19.77] |
| 4 days - 2 days | -1.33 [-15.23; 12.58] |
| 4 days - 3 days | -6.23 [-21.24; 8.78] |

*Table S5: Complete model selection analysing survival of greater snow geese marked in spring 2009 in relation to several covariates. Covariates include stress-induced corticosterone level at banding (CORT, continuous individual covariate), body condition (COND1 : condition at banding; COND2 : condition at release; both as continuous individual covariate), food treatment (FOOD: treatment fed/unfed/control), number of days spent in captivity (DAYS : 0, 2, 3 and 4 days), and time (t, in years; t(1): first year after release; t(2:13):constant for years 2 to 13; t(2_13): time varying for years 2 to 13). K = number of parameters, ΔQAICc = difference in QAICc between the current and the top-ranked model within each model selection. Three separate model selection done sequentially are presented and are separated by the grey lines. Models in bold were preferred and were used to go to the next model selection.*

| **Model name** | **Survival** | **Recapture** | **Recovery** | **K** | **Deviance** | **QAICc** | **ΔQAICc** |
| --- | --- | --- | --- | --- | --- | --- | --- |
| Initial model selection for recovery and reobservation probabilities (event matrix) | | | | | | | |
| **MS1** | **General model** | **Constant** | **Constant** | **53** | **2826.46** | **2613.40** | **0** |
| MS2 | General model | t | Constant | 64 | 2800.65 | 2615.42 | 2.03 |
| MS3 | General model | Constant | t | 64 | 2801.07 | 2615.79 | 2.40 |
| MS4 | General model | t | t | 76 | 2777.90 | 2623.11 | 9.71 |
| Model selection for survival probability (transition matrix) | | | | | | | |
| **MS5** | **t(1).FOOD+t(2:13)** | **Constant** | **Constant** | **6** | **2872.35** | **2553.99** | **0** |
| **MS6** | **t(1).[FOOD+COND1]+t(2:13)** | **Constant** | **Constant** | **7** | **2871.17** | **2554.98** | **0.99** |
| **MS7** | **t(1).[CORT+FOOD]+t(2:13)** | **Constant** | **Constant** | **7** | **2871.85** | **2555.65** | **1.58** |
| MS8 | t(1).COND2+t(2:13) | Constant | Constant | 5 | 2877.85 | 2556.83 | 2.84 |
| MS9 | t(1).[CORT+COND2]+t(2:13) | Constant | Constant | 6 | 2877.54 | 2558.58 | 4.59 |
| MS10 | t(1).COND1+t(2:13) | Constant | Constant | 5 | 2880.03 | 2558.76 | 4.77 |
| MS11 | t(1).CORT+t(2:13) | Constant | Constant | 5 | 2880.16 | 2558.88 | 4.89 |
| MS12 | t(1).[FOOD.COND2]+t(2:13) | Constant | Constant | 7 | 2876.46 | 2559.65 | 5.66 |
| MS13 | t(1).[CORT.DAYS]+t(2:13) | Constant | Constant | 8 | 2874.48 | 2559.94 | 5.94 |
| MS14 | t(1).[CORT+COND1]+t(2:13) | Constant | Constant | 6 | 2879.57 | 2560.38 | 6.39 |
| MS15 | t(1).[FOOD.COND1]+t(2:13) | Constant | Constant | 7 | 2877.34 | 2560.43 | 6.44 |
| MS16 | t(1).[CORT+DAYS]+t(2:13) | Constant | Constant | 8 | 2876.41 | 2561.64 | 7.65 |
| MS17 | Constant | Constant | Constant | 3 | 2889.10 | 2562.75 | 8.76 |
| MS18 | t(1).[CORT.FOOD]+t(2:13) | Constant | Constant | 7 | 2880.33 | 2563.10 | 9.09 |
| MS19 | t(1).FOOD+t(2_13) | Constant | Constant | 16 | 2860.48 | 2563.96 | 9.97 |
| MS20 | t(1).[CORT+FOOD]+t(2_13) | Constant | Constant | 17 | 2860.00 | 2565.60 | 12.71 |
| MS21 | t | Constant | Constant | 14 | 2869.23 | 2567.57 | 13.58 |
| MS22 | t(1).CORT+t(2_13) | Constant | Constant | 15 | 2868.27 | 2568.78 | 14.79 |
| MS23 | t(1).[CORT.FOOD]+t(2_13) | Constant | Constant | 17 | 28678.43 | 2573.07 | 20.18 |
| Final model selection on best models | | | | | | | |
| **MS24** | **t(1).FOOD+t(2:13)** | **t** | **Constant** | **17** | **2845.63** | **2552.89** | **0** |
| MS25 | t(1).FOOD+t(2:13) | t | t | 24 | 2835.31 | 2558.36 | 5.48 |
| MS26 | t(1).FOOD+t(2:13) | Constant | t | 17 | 2858.42 | 2564.21 | 11.32 |
| **MS27** | **t(1).[FOOD+COND1]+t(2:13)** | **t** | **Constant** | **18** | **2844.50** | **2553.96** | **1.07** |
| MS28 | t(1).[FOOD+COND1]+t(2:13) | Constant | t | 18 | 2857.23 | 2565.22 | 12.34 |
| MS29 | t(1).[FOOD+COND1]+t(2:13) | t | t | 29 | 2835.03 | 2568.69 | 15.80 |
| **MS30** | **t(1).[CORT+FOOD]+t(2:13)** | **t** | **Constant** | **18** | **2844.50** | **2553.96** | **1.08** |
| MS31 | t(1).[CORT+FOOD]+t(2:13) | Constant | t | 18 | 2857.91 | 2565.82 | 12.94 |
| MS32 | t(1).[CORT+FOOD]+t(2:13) | t | t | 29 | 2835.03 | 2568.69 | 15.80 |
| MS33 | i+t(1).CORT+t(2:13) | t | Constant | 16 | 2853.54 | 2557.82 | 4.93 |
| MS34 | t(1).COND1+t(2:13) | t | Constant | 16 | 2853.18 | 2557.49 | 4.61 |
| MS35 | t(1).COND2+t(2:13) | t | Constant | 16 | 2850.98 | 2555.55 | 2.66 |
| MS36 | Constant | t | Constant | 14 | 2855.77 | 2555.66 | 2.77 |

| A  *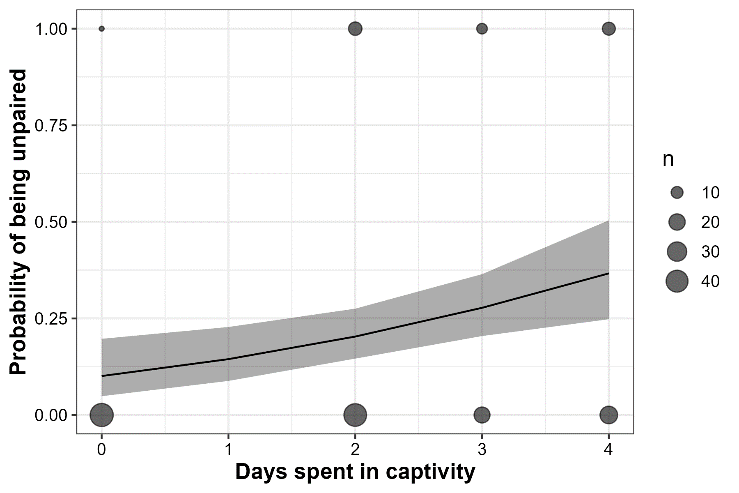* | B  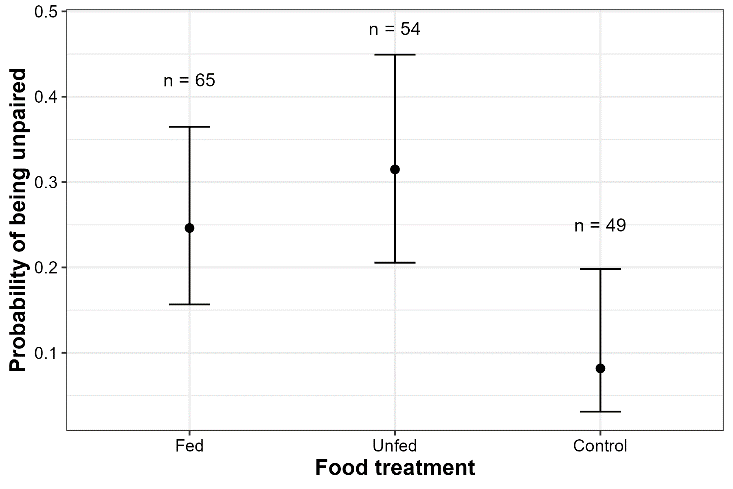 |
| --- | --- |
| C  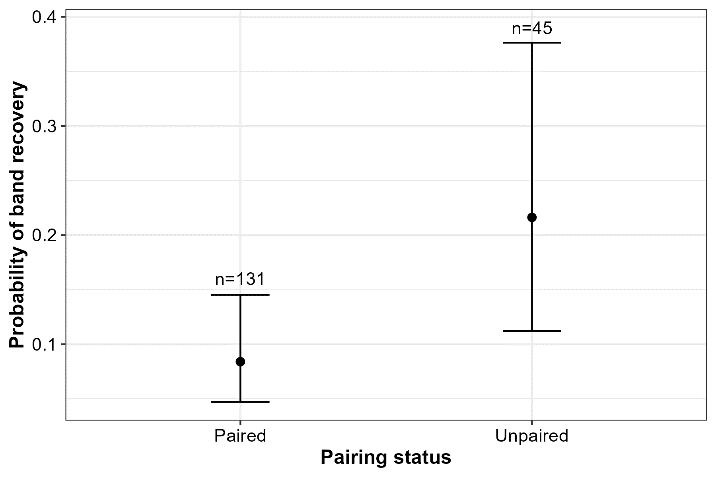 | D  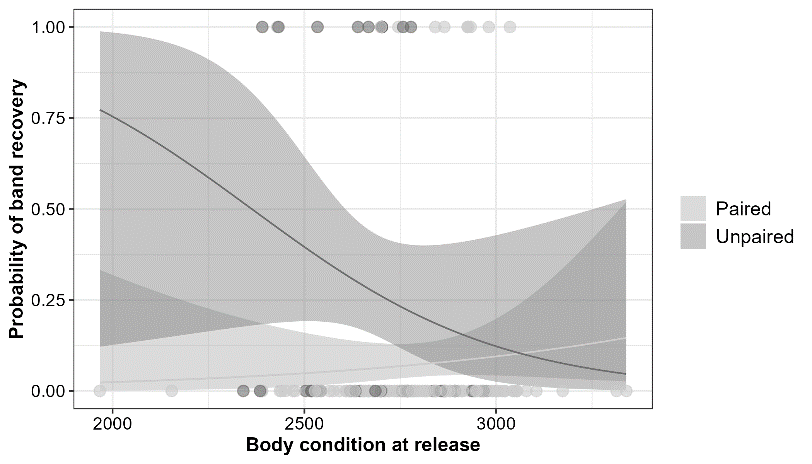 |
| *Figure S6: A: Probability of observing a marked females without a mate (i.e. unpaired) in autumn depending on the number of days spent in captivity in spring (*$\beta$ *= 0.33 [0.13, 0.54]). B: Probability of observing a marked and unpaired female in autumn as a function of the food treatment in spring (*$\beta_{fed/unfed}$*= 0.34 [-0.46, 1.15]). C: Probability of band recovery in the first year after marking of paired and unpaired females in autumn (*$\beta_{status}$*= 13.9 [-1.2, 29.9]) D: Relationship between the probability of band recovery in the first year after marking and body condition at release for paired and unpaired females observed in autumn (*$\beta_{status:condition}$ *= -0.005 [-0.01, 9.80e-04]). The parameters estimates are presented with 95% confidence intervals in all four panels.* | |
